# The reference intervals for thyroid hormones: A four year investigation in Chinese population

**DOI:** 10.3389/fendo.2022.1046381

**Published:** 2023-01-06

**Authors:** Tiancheng Xie, Mingchuan Su, Jie Feng, Xiaoying Pan, Chuan Wang, Tian Tang

**Affiliations:** ^1^ West China School of Public Health and West China Fourth Hospital, Sichuan University, Chengdu, Sichuan, China; ^2^ West China-PUMC C.C. Chen Institute of Health, Sichuan University, Chengdu, Sichuan, China

**Keywords:** thyroid hormones, reference intervals, age, sex, Chinese population

## Abstract

**Introduction:**

The reference intervals (RIs) are of great importance for physicans to determine whether or not an individual is healthy. However, many clinical laboratories in China still adopted the default RI provided by the manufacturers; and these “uncalibrated” RIs might lead to the misdiagnosis of diseases. In the present study, we enroll reference people with the purpose of determining the RIs of serum triiodothyronine (T3), thyroxine (T4), free triiodothyronine (FT3), free thyroxine (FT4) and thyroid-stimulating hormone (TSH) in Chinese population, and explore the possible roles of age and sex on the levels of biomarkers.

**Methods:**

Serum samples from 66,609 individuals who met the inclusion criteria were analyzed using an Roche Cobas E 601 hormone analyzer. The dynamic trends of biomarker were visually assessed by their concentrations over age and sex. Specific partitions were determined by the method of Harris and Boyd. RIs, corresponding to the 2.5th and 97.5th percentiles, as well as the 0.5th, 25th, 50th, 75th and 99.5th percentiles were calculated for each reference partition using a non-parametric rank approach.

**Results:**

The serum level of T3, T4, FT4 or TSH showed a right-skewed distribution in both males and females while FT3 presented an approximate normal distribution. Females had a higher mode value of serum T3 or T4, but a lower mode value of serum TSH, FT3 or FT4. All five biomarkers did not need age partitioning according to the approach of harris and boyd, while T3 and FT3 need sex partitioning.

**Conclusions:**

The present study not only determined the age- and sex-specific trends of the five thyroid hormones, but provided sex-stratified RIs for T3 and FT3, valuably contributing to the current literature and timely evaluation of thyroid health and disease.

## 1 Introduction

In China, approximately 40% of adults (≥18 years of age) suffer from thyroid-related diseases, and subclinical hypothyroidism accounts for most of these cases (1). As the symptoms of patients with subclinical thyroid disease are not apparent, biomarkers such as serum triiodothyronine (T3), thyroxine (T4), free triiodothyronine (FT3), free thyroxine (FT4) and thyroid-stimulating hormone (TSH) are used to assist in the diagnosis of thyroid diseases.

Clinically, reference intervals (RIs) are used to determine whether the level of a biomarker is within the normal range, and the use of inappropriate RIs as judgment criteria may lead to serious consequences. For example, Droke et al. suggested that the use of inadequate serum ferritin reference intervals caused an underdiagnosis (>15%) of iron deficiency in low-income children (2). In the Netherlands, using the TSH reference interval recommended by the American Thyroid Association for pregnant women caused 8.6% and 4.9% of pregnant women with normal TSH levels to be misdiagnosed in the first and second trimesters, respectively (3, 4).

Currently, most clinical laboratories in China adopt the default RIs from manufacturers. These RIs, however, were developed on limited samples and do not reflect the actual values of biomarkers in specific populations. To improve screening performance, the American Thyroid Association (ATA) and the National Academy of Clinical Biochemistry (NACB) published guidelines in 2017 suggesting that clinical laboratories should establish their own RIs based on the screened populations (5, 6). Considering this, we launched a cross-sectional study in 2016. The goal of such an investigation was to determine the baseline levels of serum TSH, T3, FT3, T4 and FT4 in a population of healthy Chinese individuals and explore the possible roles of age and sex on the levels of biomarkers. In this pilot study, we reported the dynamic trends of TSH, T3, FT3, T4 and FT4 according to age and sex and developed age- and/or sex-stratified RIs.

## 2 Materials & methods

### 2.1 Study subjects

Individuals aged 22 to 70 years who had a physical exam at West China Fourth Hospital (WCFH) between August 2016 and September 2020 were enrolled in this study. The physical conditions of the study participants were assessed by licensed medical practitioners based on their questionnaire responses and physical exam reports. Subjects with visible or palpable thyroid tumors were excluded, as were breastfeeding or pregnant women, individuals with pituitary disorders, hypothyroidism, hyperthyroidism, a family history of thyroid disease, and those taking medications that affect thyroid function (**Supplementary Table S1**).

### 2.2 Participating laboratory

The biochemical laboratory of the West China Fourth Hospital (BLWCFH) was the participating laboratory. BLWCFH passed multiple rounds of external quality assessments (EQA) organized by the National Center for Clinical Laboratories (NCCL) since 2012. The detail of the NCCL EQA procedure included the following: the NCCL distributed two batches of quality control products containing low, median and high concentration of certain target analytes to each participating laboratory every year; participating laboratories were required to test all quality control products and return the results to the NCCL. The allowable deviation for any test analyte in the NCCL was ±25% from the median value of the peer group laboratories, while the deviation for TSH, T3, T4, FT3 or FT4 in BLWCFH was less than 8%. The EQA results of BLWCFH from 2016 to 2020 are listed in **Supplementary Table S2**.

Except for the EQA, BLWCFH also performed strict internal quality control (IQC) as follows (1): control products containing high and low levels of target analytes were measured every day for 20 consecutive days; and (2) all test values were between the mean ± 2 SD, while the coefficient of variation (CV) was limited to <8%. Over the 4-year period during which samples were tested, 5 batches of control products were used to monitor the performance of the testing equipment. The overall imprecision for each analyte is listed in **Supplementary Table S3**.

### 2.3 Sample acquisition and testing

For all study participants, 8 hours of fasting were required before blood sampling. In brief, 3 ml of blood was collected from each subject using a vacutainer blood collection tube (Sanfeng Medical Co., Ltd., Chongqing, China). After incubation at room temperature for 10 min, the serum was isolated by centrifugation at 3,000 rpm for 10 minutes. The levels of T3, T4, FT3, FT4 and TSH were determined using a Cobas E 601 automatic electro-chemiluminescence immunoassay analyzer (Roche Diagnostics, Shanghai, China) with the corresponding Elecsys^®^ detection Kit. The time interval between sampling and testing was <60 min. The limits of detection (LOD)/quantification (LOQ) are listed in **Supplementary Table S4**.

### 2.4 Statistical analysis

Data were analyzed in accordance with CLSI C28-A3 guidelines (7). Statistical analysis was implemented using R studio Ver. 4.0.5 or EXCEL Ver. 16.65. In brief, the frequency histograms of each biomarker were used to visually inspect the data. Outliers were identified using Tukey’s tests and removed. The normality of the data was determined by the Shapiro−Wilk test. The RIs of each biomarker were partitioned by the Harris & Boyd approach (7); if the results did not suggest partitioning, the data were combined and re-estimated. The nonparametric rank method was used to calculate the 0.5th, 2.5th, 25th, 50th, 75th, 97.5th and 99.5th percentiles of each reference partition. The central 95% interval, corresponding to the range between the 2.5th and 97.5th percentiles, was defined as the RI. For each RI, the 90% confidence intervals were calculated for the end points.

## 3 Results

### 3.1 The sample size of each biomarker

A total of 66,609 adults (22 to 70 years old) who met the inclusion criteria were included in the study analysis. As the study subjects were allowed to choose their health check packages and different packages included varying numbers of tested thyroid hormones, it is not surprising that the data size for serum levels of TSH, T3, T4, FT3 and FT4 are different. Specifically, the TSH level was determined for all subjects. While the serum level of T3 and T4 were measured for approximately 54,000 subjects. In comparison, only 34,000 subjects choose to take all five thyroid hormones. The sample size of each analyte and the number of outliers removed are listed in **Table 1**.

**Table 1:** Sample information.

Analytes	Total No. of samples	No. of samples been excluded & exlusion rate (%)
**T3**	54398	410 (0.75)
**T4**	54399	324 (0.60)
**FT3**	34156	777 (2.27)
**FT4**	33992	603 (1.77)
**TSH**	66609	3603 (5.41)

T3, triiodothyronine; T4, thyroxine; FT3, free triiodothyronine; FT4, free thyroxine; TSH, thyroid-stimulating hormone.

### 3.2 The distribution of biomarkers

**Figure 1:**
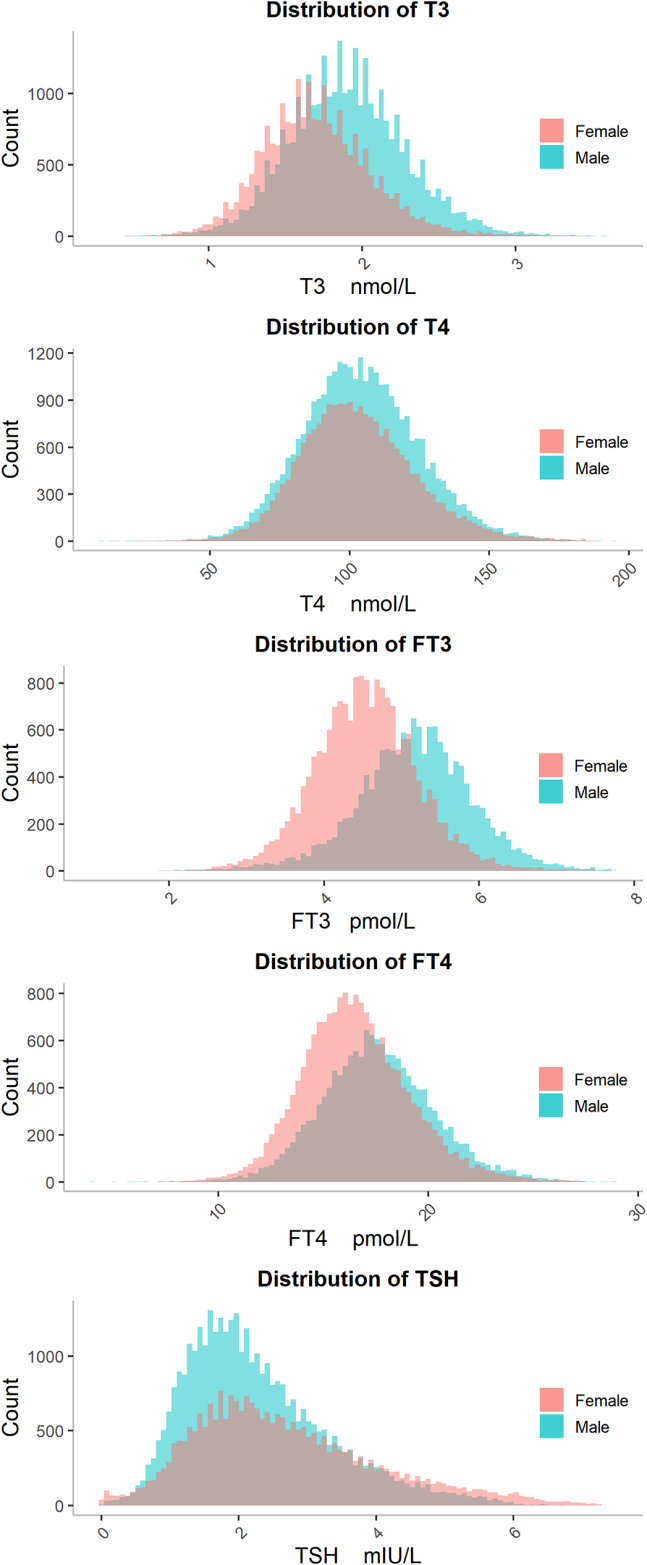
The distributions of T3, T4, FT3, FT4 and TSH. The distributions of five thyroid hormones are shown separately for males (blue bars) and females (red bars). Abbreviations are listed in the legend of **Table 1**.

As indicated in **Figure 1**, the serum level of T3 in females and the serum level of TSH in both males and females showed a right-skewed distribution, while T4, FT3 and FT4 showed an approximately normal distribution. Females had a higher mode value of TSH (2.15 mIU/L), whereas males had a higher mode values of T3 (1.87 nM), T4 (107.0 nM), FT3 (5.14 pM), and FT4 (17.90 pM) (**Table 2**). The skewness value for each biomarker by sex is listed in **Table 2**. The distribution of biomarker values according to various age groups is listed in **Supplementary Figure S6–S10**.

**Table 2:** The skewness values and mode values for T3, T4, FT3, FT4, and TSH by sex.

Analytes	Gender	skew values	mode values
**T3**	male	0.232	1.87 nM
	female	0.578	1.57 nM
**T4**	male	0.228	107.00 nM
	female	0.427	100.50 nM
**FT3**	male	0.262	5.14 pM
	female	0.220	4.48 pM
**FT4**	male	0.175	17.90 pM
	female	0.418	15.20 pM
**TSH**	male	0.875	1.75 mIU/L
	female	0.716	2.15 mIU/L

Abbreviations are listed in the legend of **Table 1**.

### 3.3 The change in biomarkers according to age and sex

**Figure 2:**
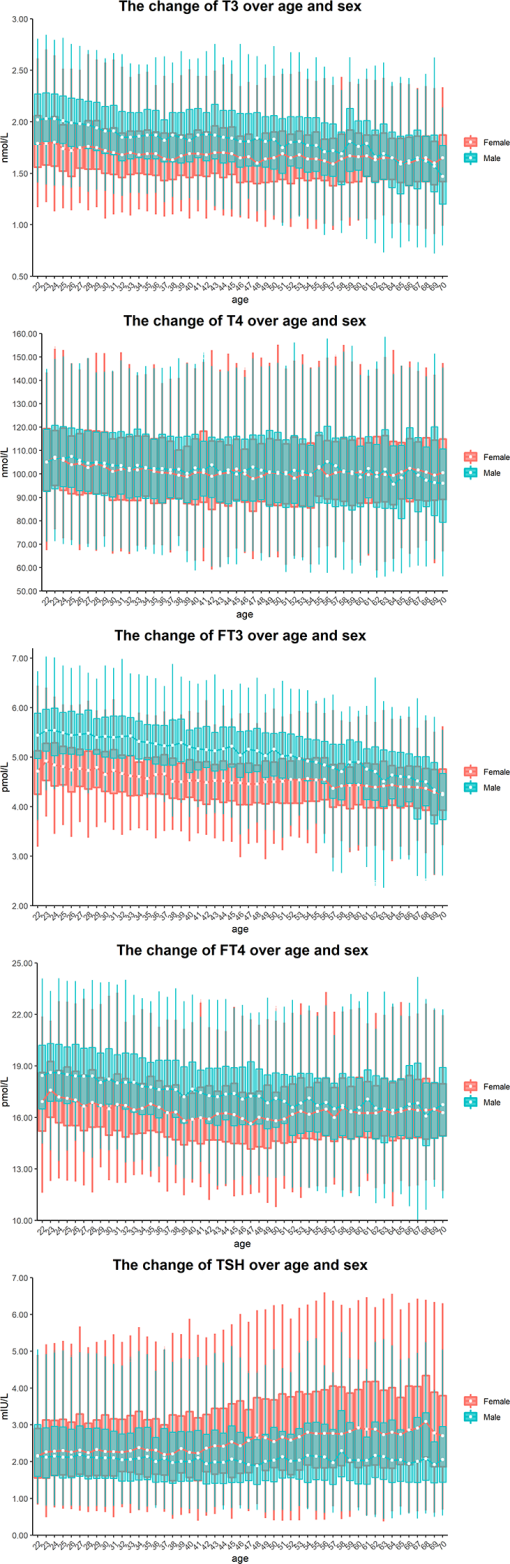
Sex and age-related dynamic changes of T3, T4, FT3, FT4 and TSH. Data of male and female partitions are shown in blue and red boxes with whiskers, respectively. The boxes extend from the 25^th^ to the 75^th^ percentile, with whiskers extending to the 2.5^th^ or 97.5^th^ percentile. Medians are shown as white circles in the body of boxes, and are linked with blue (male) or red (female) line to shown the dynamics. Abbreviations are listed in the legend of **Table 1**.

As shown in **Figure 2**, the median of T3 decreased in males and remained at a steady state in females according to age. In contrast, the median of FT3 in all male partitions was higher than that in female partitions, except for the age group of 70 years old. The dynamic trend of serum FT4 was similar to that of serum FT3, except that the median of FT4 in males aged 68 years was lower than that of females of the same age. Although the level of serum T4 did not change with age or sex, the median and interquartile range (IQR) of TSH increased with age in females.

### 3.4 The proposed RIs versus the default RIs

As the Harris & Boyd method did not suggest any age and/or sex partitioning for T4, FT4 and TSH but suggested sex partitioning for both T3 and FT3, the estimated RIs for T4, FT4 and TSH were [65.90-147.5] nM, [12.11-22.82] pM and [0.683-5.45] mIU/L, respectively (**Table 3**). The RIs of T3 and FT3 for males were [1.20-2.70] nM and [3.54-6.63] pM, respectively (**Table 3**), while the RIs of T3 and FT3 for females were [1.06-2.49] nM and [3.24-5.93] pM, respectively (**Table 3**). For each RI, the 90% confidence intervals for the end points calculated by the bootstrap resampling method are shown in **Table 4**. The details of the other percentiles for each biomarker are listed in **Supplementary Table S5**.

**Table 3:** RIs provided by the manufacturer versus the RIs we proposed.

Analytes	Gender	RIs provided by the manufacturer	RIs we proposed
**T3**	male	(1.3-3.1) nM	(1.20-2.70) nM
	female		(1.06-2.49) nM
**T4**	unisex	(66-181) nM	(65.90-147.5) nM
**FT3**	male	(3.1-6.8) pM	(3.54-6.63) pM
	female		(3.24-5.93) pM
**FT4**	unisex	(12-22) pM	(12.11-22.82) pM
**TSH**	unisex	(0.270~4.20) mIU/L	(0.683-5.45) mIU/L

Abbreviations are listed in the legend of **Table 1**.

**Table 4:** The RIs (95% CI) for T3, T4, FT3, FT4, and TSH.

Analytes	Gender	Lower limit (2.5^th^) & 90% CI	Upper limit (97.5^th^) & 90% CI	No. of samples
**T3**	male	1.20 (1.19-1.21) nM	2.70 (2.68-2.71) nM	30932
	female	1.06 (1.05-1.07) nM	2.49(2.47-2.50) nM	23056
**T4**	unisex	65.90 (65.58-66.20) nM	147.5 (147.1-148.1) nM	54075
**FT3**	male	3.54 (3.52-3.59) pM	6.63 (6.59-6.65) pM	14935
	female	3.24 (3.22-3.27) pM	5.93 (5.91-5.96) pM	18444
**FT4**	unisex	12.11 (12.06-12.17) pM	22.82 (22.72-22.90) pM	33389
**TSH**	unisex	0.683 (0.671-0.695) mIU/L	5.45 (5.42-5.48) mIU/L	63006

Abbreviations are listed in the legend of **Table 1**.

## 4 Discussion

Estimating RIs has never been an easy job, but determining the reference partition is even tougher. Although several reports published age- and/or sex-stratified RIs for thyroid hormones, they did not interpret how they partitioned the reference groups. For example, Vadiveloo et al. divided the reference population into 8 age groups and established age-specific RIs for serum TSH. In their study, less information regarding the rationale for group partitioning is available (8). In another case, Yoshihara and colleagues established age- and sex-stratified RIs for serum TSH and FT4. However, again, they did not explain how they confirmed the reference partitions (9). In the present study, the reference partitions were determined by the Harris & Boyd method. The merit of using such a statistical method is that many cofactors, e.g., sample size and value distribution, could be taken into consideration when determining the reference groups to avoid any subjective preference (10, 11).

The actual distribution of TSH, T3, T4, FT3 and FT4 in serum samples was assessed by estimating the 0.5th, 2.5th, 25th, 50th, 75th, 97.5th and 99.5th percentiles in each reference partition. As shown in **Figure 2**, the median TSH level was lower in males than in females. This result is consistent with the findings reported previously (12, 13). Unlike TSH, the median level of FT4 was higher in males than in females. This may be attributed to the differential expression of DIO1, LHX3 and FOXE1 between men and women. Such genes were demonstrated to affect the level of FT4 *via* the hypothalamic-pituitary-thyroid axis (14). As the activity of 5’-deiodinase deiodinase found in young males was strong (15), it is not surprising that the levels of T3 and FT3 were higher in adolescent males (20–24 years old) since 5’-deiodinase is able to convert T4 to T3 (16). Regarding the level of T4, previous studies reported a higher level of T4 in males, which is in line with our study (17, 18). The TSH level barely changed over age in males but increased steadily in females from the age of 40 to 68 years (**Figure 2**). One report attributed this TSH increase either to the decreased activity of TSH in middle-aged women or to a decreased sensitivity of TSH to the negative feedback of thyroid hormones (19), while another report attributed this to the increased uptake of iodine or the accelerated organ failure of thyroid tissues with age in females (20).

Although elderly individuals are more likely to suffer from thyroid diseases that affect their life expectancy and increase the burden on society as a whole (1, 21), few studies have investigated the baseline levels and dynamic trends of thyroid hormones in elderly individuals. Our study closed this gap by including elderly individuals aged 60 to 70 years. As people age, the levels of T3 and FT3 tend to decrease, while T4 and FT4 levels remain relatively stable (**Figure 2**). In a previous study, the levels of FT3 and T3 were found to be higher in people aged 18-50 years compared to those aged 51-90 years. Specifically, FT3 levels were about 6%~7% higher in the younger group, while T3 levels were about 8%~12% higher (22). Additionally, the study found no correlation between age and the level of FT4 and T4 (22), which is consistent with the data of the present study.

The most noteworthy finding in this study is that we captured the dynamic trends of TSH, T3, FT3, T4 and FT4 over age and sex. Comparing to the RIs provided by the manufacturers, the RIs that we proposed had a lower endpoint for both T3 and T4, which aligns with several studies (23, 24). Furthermore, a large sample size allowed us to determine a more precise reference interval (RI) for FT3 than the previous studies (25, 26). In a recent investigation, Wang and colleges showed that the reference range of TSH in healthy Chinese adults was (0.67-7.87) mIU/L (27). In contrast, our RIs presented a lower upper limit. Differential results could be due to the sample size, age and ethnicity of the study population.

It is important to show the limitations of our study. As the inclusion and exclusion criteria for the healthy population were not rigorous, there may have been a mix of false-positive and false negative cases, which might impair the accuracy of thyroid hormone RIs. However, since tens of thousands of samples were included in this study, such an error can be largely ignored. Although our study showed statistically significant sex differences in T3 and FT3 levels, some differences may be due to the large sample sizes and are not clinically significant. Other limitations are that we did not address whether additional cofactors such as iodine intake, ethnicity, climate, and season changed. That is because the submitted data from the participating laboratory did not include more detailed information. In addition, the concentration of thyroid hormones outside of the RIs does not necessarily indicate the presence of disease.

## 5 Conclusions

In conclusion, this study established age- and sex-stratified reference intervals for five thyroid function indicators based on CLSI EP28-A3c. Most importantly, our data provided strong evidence regarding the age- and sex-specific trends in thyroid hormones based on a large number of observations. Thus, these data made a valuable contribution to the current literature and could be important for the early assessment of disease.

## Data availability statement

The original contributions presented in the study are included in the article/**Supplementary Material**. Further inquiries can be directed to the corresponding author.

## Ethics statement

The studies involving human participants were reviewed and approved by the Medical Ethics Committee of West China Fourth Hospital of Sichuan University. The patients/participants provided their written informed consent to participate in this study.

## Author contributions

CW, TT, MS, and TX contributed to the conception and designed of the project. Recruitment of subjects, sample collection and measurements were conducted by TX, MS, JF, and XP. The initial draft of the manuscript was written by TX and MS. CW and TT reviewed the draft manuscript and made comments and suggestions. TX and MS made revisions to the manuscript. All authors contributed to the article and approved the submitted version.
