# Supplementary material for: The reference intervals for thyroid hormones: A four year investigation in Chinese population
Source: Front Endocrinol (Lausanne). 2023 Jan 6;13:1046381. doi: 10.3389/fendo.2022.1046381 (PMC9852975; doi:10.3389/fendo.2022.1046381)

Table S1. Medications affecting thyroid function

**Drugs that decrease TSH secretion**
Dopamine, Glucocorticoids, Octreotide
**Drugs that alter thyroid hormone secretion**
Lithium, Iodide, Amiodarone, Aminoglutethimide, Iodide, Amiodarone
**Drugs that decrease T4 absorption**
Colestipol, Cholestyramine, Aluminum hydroxide, Ferrous sulfate, Sucralfate
**Drugs that alter T4 and T3 transport in serum**
Estrogens, Tamoxifen, Heroin, Methadone, Mitotane, Fluorouracil, Androgens, Anabolic steroids (e.g., danazol), Slow-release nicotinic acid, Glucocorticoids, Furosemide, Fenclofenac, Mefenamic acid, Salicylates
**Drugs that alter T4 and T3 metabolism**
Phenobarbital, Rifampin, Phenytoin, Carbamazepine, Propylthiouracil, Amiodarone, Beta-adrenergic–antagonist drugs, Glucocorticoids
**Cytokines**
Interferon alfa, Interleukin-2
**Iodine-Containing Medications and Radiographic Contrast Agents**
Iophen, Organidin (iodinated glycerol), Par Glycerol, R-Gen, Potassium iodide, Iodo-Niacin, Mudrane, Elixophyllin-KI (theophylline) elixir, Iophylline, Iodoquinol, Povidone–iodine, Clioquinol cream, Povidone–iodine , Iopanoic acid, Ipodate sodium, Intravenous preparations

Abbreviation: TSH, thyroid-stimulating hormone; T3, triiodothyronine; T4, thyroxine.

Table S2. The external quality assessment results of BLWCFH during the period of 2016 to 2020

Time		Deviation of T3(%)	Deviation of T4(%)	Deviation of FT3(%)	Deviation of FT4(%)	Deviation of TSH（%）
**First half of 2016**	TA_{low}	-3.89	1.55	6.15	0.59	-4.38
	TA_{high}	-0.77	1.48	-1.38	0.29	-5.47
**Second half of 2016**	TA_{low}	2.35	-2.79	-6.67	1.29	-5.83
	TA_{high}	2.89	-4.32	-4.74	1.37	0.71
**First half of 2017**	TA_{low}	3.85	-7.87	-6.16	-3.57	1.09
	TA_{high}	0.54	-6.58	-2.95	-4.33	0.46
**Second half of 2017**	TA_{low}	-7.10	-3.08	-5.45	1.81	0.91
	TA_{high}	-3.13	-0.80	-5.83	0.91	-3.43
**First half of 2018**	TA_{low}	-1.16	-1.15	-3.57	0.67	-4.69
	TA_{high}	-2.90	-2.14	-1.83	-2.55	-5.01
**Second half of 2018**	TA_{low}	-6.12	-6.7	-3.32	-4.27	-5.11
	TA_{high}	-5.39	-5.98	-3.57	-5.6	-4.98
**First half of 2019**	TA_{low}	5.74	-2.61	5.24	-6.38	-2.93
	TA_{high}	5.60	-1.71	3.86	-3.94	-1.28
**Second half of 2019**	TA_{low}	-7.76	2.92	3.57	-4.25	-3.76
	TA_{high}	-5.49	4.33	0.96	-5.72	-1.85
**First half of 2020**	TA_{low}	3.75	-5.38	4.69	5.77	-5.97
	TA_{high}	2.96	-3.16	3.70	4.28	-5.67
**Second half of 2020**	TA_{low}	-3.59	4.76	4.18	7.86	-2.40
	TA_{high}	-4.27	2.74	6.69	5.17	-2.61

Abbreviation: TA_{low}, target analyte at low concentration; TA_{high}, target analyte at high concentration; T3, triiodothyronine; T4, thyroxine; FT3, free triiodothyronine; FT4, free thyroxine; TSH, thyroid-stimulating hormone.

Table S3. Accumulated coefficient of variation of internal quality control

**Analytes**	2017		2018		2019		2020	
	QC_{low}	QC_{high}	QC_{low}	QC_{high}	QC_{low}	QC_{high}	QC_{low}	QC_{high}
**T3(nM)**	6.4	4.8	4.5	3.1	5.9	5.3	4.4	4.3
**T4 (nM)**	6.7	5.2	7.3	8.6	6.1	6.3	6.8	6.2
**FT3 (pM)**	6.3	5.5	6.6	5.9	5.8	5.4	6.0	6.0
**FT4 (pM)**	5.8	7.1	6.2	4.3	5.9	5.4	4.2	2.7
**TSH (mIU/L)**	7.8	7.3	6.7	6.7	4.8	4.6	4.5	5.2

Abbreviation: QC, quality control; T3, triiodothyronine; T4, thyroxine; FT3, free triiodothyronine; FT4, free thyroxine; TSH, thyroid-stimulating hormone.

Table S4. The limits of detection (LoD)/quantitation (LoQ)

Analytes	LoD	LoQ
**T3**	0.300 nM	-
**T4**	5.40 nM	-
**FT3**	0.6 pM	1.5 pM
**FT4**	0.5 pM	1.3 pM
**TSH**	0.005 mIU/L	0.005 mIU/L

Note: **"-"** means not determined; T3, triiodothyronine; T4, thyroxine; FT3, free triiodothyronine; FT4, free thyroxine; TSH, thyroid-stimulating hormone.

Table S5. The 2.5^{th}, 25th, 50th, 75th and 97.5^{th} percentiles calculated by age and sex for 5 thyroid hormones

		Male							Female						
**Analytes**	**Age**	**0.5^{th}**	**2.5^{th}**	**25th**	**50th**	**75th**	**97.5^{th}**	**99.5^{th}**	**0.5^{th}**	**2.5^{th}**	**25th**	**50th**	**75th**	**97.5^{th}**	**99.5^{th}**
T3 (nM)	22	1.26	1.41	1.78	2.02	2.27	2.81	2.99	1.03	1.17	1.56	1.79	2.06	2.62	3.04
	23	1.22	1.39	1.81	2.03	2.28	2.85	3.36	1.01	1.22	1.58	1.79	2.03	2.75	3.45
	24	1.22	1.38	1.80	2.03	2.27	2.80	3.16	0.91	1.13	1.57	1.80	2.05	2.65	3.46
	25	1.12	1.38	1.79	2.01	2.25	2.82	3.13	0.99	1.16	1.52	1.74	1.97	2.52	2.83
	26	1.19	1.36	1.76	1.99	2.23	2.76	3.18	0.96	1.14	1.47	1.72	1.99	2.52	2.98
	27	1.17	1.37	1.75	1.98	2.22	2.74	3.15	1.09	1.21	1.55	1.75	1.98	2.51	2.96
	28	1.15	1.32	1.74	1.97	2.20	2.70	3.03	0.98	1.14	1.54	1.76	2.01	2.66	3.36
	29	1.19	1.34	1.72	1.94	2.19	2.70	3.01	0.99	1.17	1.54	1.75	2.01	2.61	3.19
	30	1.12	1.33	1.69	1.91	2.15	2.62	2.88	0.90	1.06	1.50	1.72	1.93	2.59	3.10
	31	1.14	1.30	1.68	1.91	2.16	2.67	2.97	0.83	1.10	1.49	1.70	1.94	2.61	3.09
	32	1.12	1.28	1.62	1.85	2.10	2.66	3.11	1.00	1.12	1.46	1.68	1.89	2.60	3.01
	33	1.08	1.31	1.64	1.85	2.09	2.57	2.83	0.93	1.09	1.49	1.70	1.91	2.44	2.70
	34	1.13	1.31	1.65	1.86	2.09	2.56	2.77	1.03	1.15	1.50	1.68	1.91	2.47	3.21
	35	1.07	1.30	1.64	1.87	2.12	2.56	2.75	0.95	1.13	1.49	1.69	1.92	2.49	3.01
	36	1.16	1.31	1.64	1.87	2.11	2.63	2.91	0.93	1.10	1.48	1.69	1.90	2.36	2.69
	37	1.08	1.22	1.61	1.82	2.02	2.64	2.97	0.94	1.08	1.43	1.64	1.89	2.45	2.78
	38	1.09	1.27	1.61	1.87	2.09	2.58	2.93	0.98	1.10	1.44	1.63	1.89	2.38	2.76
	39	1.05	1.24	1.60	1.85	2.09	2.69	2.98	1.01	1.13	1.48	1.67	1.87	2.47	2.76
	40	1.02	1.26	1.61	1.82	2.12	2.61	2.78	0.92	1.06	1.46	1.69	1.88	2.58	3.09
	41	1.01	1.24	1.65	1.87	2.13	2.62	2.91	0.93	1.13	1.48	1.69	1.92	2.45	2.78
	42	1.11	1.24	1.62	1.88	2.09	2.69	2.92	0.92	1.06	1.47	1.68	1.90	2.45	2.96
	43	0.92	1.18	1.64	1.87	2.17	2.76	3.17	1.02	1.14	1.50	1.70	1.96	2.50	2.82
	44	0.83	1.15	1.60	1.85	2.11	2.63	2.83	1.00	1.09	1.46	1.70	1.94	2.56	3.21
	45	1.00	1.15	1.61	1.84	2.11	2.60	2.81	0.99	1.08	1.49	1.69	1.95	2.47	2.79
	46	0.97	1.09	1.56	1.81	2.08	2.76	3.26	0.89	1.07	1.41	1.65	1.86	2.35	2.82
	47	1.06	1.25	1.57	1.81	2.08	2.66	2.86	0.78	1.06	1.42	1.66	1.88	2.39	2.77
	48	0.97	1.13	1.57	1.84	2.09	2.64	2.85	0.84	1.03	1.40	1.60	1.83	2.36	2.73
	49	0.86	1.14	1.56	1.81	2.05	2.56	2.75	0.86	0.98	1.41	1.64	1.89	2.43	2.56
	50	0.88	1.09	1.54	1.83	2.06	2.74	3.05	0.84	1.05	1.42	1.65	1.90	2.45	2.71
	51	0.69	0.99	1.50	1.75	2.03	2.52	3.01	0.82	1.02	1.44	1.69	1.91	2.47	3.03
	52	0.93	1.06	1.57	1.80	2.05	2.68	3.04	0.89	0.98	1.40	1.65	1.88	2.47	2.88
	53	0.81	1.08	1.55	1.81	2.05	2.68	3.02	0.78	1.09	1.45	1.68	1.91	2.49	2.78
	54	0.80	1.06	1.51	1.77	2.04	2.52	2.71	0.79	0.96	1.43	1.64	1.87	2.42	2.80
	55	1.05	1.05	1.52	1.77	2.04	2.63	3.09	0.77	0.96	1.45	1.64	1.90	2.40	2.81
	56	0.46	0.90	1.48	1.71	1.99	2.54	2.91	0.83	1.01	1.43	1.62	1.83	2.44	2.89
	57	0.71	0.99	1.47	1.72	1.98	2.44	2.58	0.68	0.95	1.38	1.59	1.84	2.36	2.91
	58	0.79	0.92	1.39	1.69	1.90	2.31	2.40	0.85	1.04	1.42	1.64	1.88	2.44	2.67
	59	0.72	1.07	1.52	1.81	2.13	2.63	2.84	0.78	0.99	1.41	1.67	1.92	2.39	2.70
	60	0.86	1.05	1.53	1.76	2.01	2.42	2.67	0.89	1.04	1.42	1.66	1.89	2.37	3.02
	61	0.65	0.86	1.47	1.79	2.01	2.52	2.77	0.73	1.06	1.47	1.67	1.87	2.42	2.83

	62	0.55	0.82	1.41	1.66	1.92	2.55	2.71	0.89	1.05	1.42	1.63	1.87	2.41	2.83
	63	0.55	0.73	1.44	1.69	1.98	2.58	2.90	0.87	1.09	1.44	1.65	1.89	2.43	2.81
	64	0.72	0.87	1.36	1.65	1.90	2.39	2.67	0.86	1.01	1.44	1.65	1.84	2.35	2.63
	65	0.59	0.78	1.30	1.59	1.86	2.44	2.56	0.82	1.02	1.41	1.61	1.84	2.38	2.65
	66	0.62	0.84	1.38	1.62	1.87	2.43	2.57	0.85	1.00	1.38	1.60	1.85	2.37	2.81
	67	0.66	0.78	1.35	1.65	1.92	2.32	2.41	0.72	0.99	1.42	1.63	1.86	2.33	2.52
	68	0.58	0.78	1.42	1.62	1.86	2.47	3.12	0.84	0.96	1.42	1.65	1.86	2.35	2.69
	69	0.56	0.72	1.32	1.57	1.87	2.63	2.73	0.68	0.91	1.39	1.60	1.81	2.33	2.55
	70	0.71	0.80	1.20	1.47	1.77	2.14	2.60	0.74	0.99	1.42	1.65	1.87	2.34	2.53
T4 (nM)	22	60.26	71.06	92.53	105.10	119.13	145.07	162.70	48.53	67.43	92.79	105.05	119.40	143.58	161.07
	23	62.03	71.28	93.63	106.70	120.80	149.34	171.12	65.71	76.35	95.14	107.10	118.50	154.54	166.13
	24	61.81	70.10	94.58	106.60	120.20	150.46	164.43	64.29	72.53	93.04	105.80	119.55	153.26	174.09
	25	62.29	69.63	94.26	107.50	119.70	147.45	167.45	60.93	71.76	91.55	104.00	116.25	147.51	163.38
	26	59.45	69.86	92.86	105.80	119.00	147.50	164.95	58.27	67.46	91.11	104.50	117.20	144.88	163.36
	27	59.82	68.78	92.64	104.60	118.33	149.73	163.68	51.86	70.14	91.67	102.70	118.65	149.71	166.80
	28	57.18	67.62	91.33	104.95	117.90	143.60	164.30	53.32	67.20	91.44	104.30	118.35	152.00	171.70
	29	59.16	68.89	90.78	104.60	117.80	143.82	160.63	68.05	73.35	91.73	103.20	117.90	151.88	170.65
	30	56.07	67.02	90.17	103.70	117.73	144.30	160.63	50.69	65.93	88.75	101.10	115.00	143.95	166.64
	31	61.34	68.18	91.42	103.60	118.05	148.70	162.53	57.14	66.86	88.97	102.00	115.55	152.20	174.60
	32	56.82	66.79	89.70	101.55	116.98	145.37	160.91	51.43	65.72	88.72	102.40	115.90	147.13	160.33
	33	55.83	66.92	90.25	103.70	119.25	143.68	152.67	59.85	69.35	88.72	101.75	116.20	142.35	157.95
	34	62.34	67.74	89.55	102.50	117.10	146.34	162.00	56.83	68.88	90.50	102.70	116.20	142.82	155.46
	35	57.58	65.87	89.39	102.30	114.90	145.40	159.16	61.54	69.78	87.48	101.10	114.95	147.08	167.53
	36	54.20	65.32	87.62	102.25	116.95	145.53	160.42	61.34	69.53	87.55	100.60	115.53	138.99	165.22
	37	57.76	67.44	89.92	101.85	116.10	146.15	159.04	57.02	66.39	89.38	100.45	113.98	140.88	160.74
	38	62.02	69.26	89.26	102.00	115.78	149.69	161.96	66.02	73.43	89.64	99.43	110.20	144.17	175.82
	39	54.96	62.35	87.71	100.20	116.15	147.40	160.73	59.47	67.08	87.38	98.79	111.80	147.81	163.09
	40	50.92	58.77	89.20	102.60	117.00	147.20	162.57	51.42	63.00	86.87	100.65	115.00	145.40	168.56
	41	58.33	68.48	90.53	101.75	113.93	151.93	167.78	41.14	62.66	87.90	100.55	118.25	148.07	165.46
	42	34.84	60.43	89.97	103.85	115.73	146.32	163.25	46.26	59.15	84.85	99.00	114.10	153.20	178.66
	43	34.15	60.11	88.02	100.70	114.90	143.99	155.34	53.87	60.33	87.63	100.15	113.83	144.83	166.37
	44	44.78	61.48	87.30	101.70	115.80	144.14	165.60	53.25	64.49	86.39	100.60	112.38	151.67	176.21
	45	56.29	61.41	88.40	99.97	116.10	145.64	162.51	61.92	68.09	89.04	102.10	114.80	147.60	171.20
	46	58.52	66.76	88.69	101.40	114.90	141.60	166.50	49.49	66.22	87.99	100.04	110.63	145.51	153.11
	47	58.21	67.02	88.39	102.90	116.68	149.63	157.50	57.35	63.60	84.07	97.98	114.85	152.19	161.44
	48	53.38	62.94	89.10	101.65	116.63	146.09	166.22	57.29	66.23	88.21	98.97	112.93	149.96	179.31
	49	50.27	64.44	86.20	100.90	118.70	146.32	163.02	38.18	64.47	86.80	100.65	115.08	147.72	165.61
	50	58.71	66.87	87.77	100.70	117.85	147.67	162.34	49.83	64.41	86.51	100.20	114.50	155.50	175.10
	51	49.42	58.13	85.67	100.05	114.63	146.47	177.29	46.88	61.22	87.30	100.70	112.80	145.28	178.59
	52	50.11	63.74	87.49	101.50	116.40	156.48	163.69	52.61	64.98	86.01	98.19	115.80	148.67	160.93
	53	44.28	63.05	85.90	101.10	115.00	147.06	175.58	55.08	64.41	86.31	99.76	112.75	151.43	171.53
	54	41.38	57.79	86.12	99.80	112.30	145.28	156.76	53.31	61.36	85.46	99.52	113.20	145.76	162.21
	55	53.04	61.32	87.66	102.60	117.80	145.90	155.14	56.67	68.57	90.57	103.10	116.50	148.52	175.85
	56	37.31	57.75	87.43	105.30	120.40	158.13	166.48	56.04	69.17	89.31	99.23	114.28	149.96	171.50
	57	51.36	64.18	86.06	103.50	115.40	146.46	162.06	57.35	64.38	89.12	100.50	113.73	151.03	172.55
	58	52.79	59.00	86.38	100.85	115.73	152.48	170.11	62.77	70.91	88.32	101.25	115.10	155.41	171.52
	59	42.07	57.48	84.56	100.90	116.00	155.03	165.78	56.90	63.35	86.54	100.09	114.43	148.30	170.64

	60	53.17	60.82	86.00	98.62	111.90	142.55	163.16	52.54	62.06	87.60	101.00	115.20	147.13	163.76
	61	61.80	65.88	89.80	102.30	115.90	144.70	163.83	52.63	65.08	87.54	99.70	113.30	142.22	158.17
	62	48.55	55.70	85.83	98.94	112.65	148.35	173.96	47.48	58.79	87.88	100.50	115.98	145.16	166.44
	63	40.25	56.19	85.34	101.95	118.90	158.87	166.60	50.63	62.36	88.10	99.72	116.40	150.25	167.05
	64	43.20	58.32	85.23	95.62	112.00	142.94	158.25	50.09	63.68	86.92	99.34	113.85	153.27	165.12
	65	48.43	57.59	80.92	98.38	112.20	146.54	167.40	57.94	68.25	89.36	100.90	113.40	146.50	168.98
	66	38.00	60.13	89.50	102.40	119.80	145.73	156.66	54.69	65.84	88.20	102.40	115.10	148.34	169.24
	67	27.75	60.81	83.80	99.50	115.60	148.00	160.70	56.02	68.45	88.86	101.70	113.70	144.30	161.78
	68	39.15	60.44	86.10	97.37	111.53	141.90	159.83	62.17	71.41	88.53	100.75	115.45	145.75	163.14
	69	44.61	63.54	82.22	96.32	120.20	151.67	166.61	46.84	61.82	88.76	99.49	114.40	142.62	155.22
	70	35.76	56.26	79.36	96.01	110.70	145.68	163.21	59.97	66.91	89.12	100.50	114.88	147.65	160.25
FT3 (pM)	22	3.45	3.73	4.97	5.44	5.89	6.74	7.05	2.98	3.19	4.25	4.72	5.13	6.73	7.07
	23	3.91	4.33	5.12	5.54	5.97	7.04	7.44	3.57	3.80	4.53	4.92	5.28	6.52	6.85
	24	3.87	4.35	5.03	5.54	5.99	7.02	7.43	3.07	3.45	4.42	4.84	5.29	6.23	7.03
	25	4.18	4.40	5.07	5.49	5.90	6.86	7.39	3.05	3.63	4.44	4.81	5.22	6.07	6.32
	26	4.08	4.42	5.06	5.44	5.93	6.81	7.13	3.06	3.39	4.30	4.74	5.19	6.06	6.83
	27	3.92	4.41	5.04	5.46	5.87	6.86	7.38	3.34	3.69	4.41	4.77	5.16	6.07	6.44
	28	3.90	4.27	5.15	5.48	5.95	6.71	7.56	3.06	3.37	4.36	4.73	5.12	6.15	7.11
	29	4.13	4.41	5.06	5.40	5.78	6.59	7.30	3.29	3.59	4.39	4.79	5.15	5.95	6.47
	30	4.03	4.30	5.00	5.42	5.81	6.86	7.14	3.31	3.68	4.31	4.65	5.11	5.97	6.43
	31	3.91	4.32	4.98	5.40	5.83	6.81	7.41	2.73	3.53	4.27	4.73	5.15	6.18	6.82
	32	3.84	4.30	4.98	5.42	5.83	6.99	7.23	2.72	3.43	4.30	4.67	5.04	5.86	6.24
	33	4.01	4.22	5.03	5.43	5.80	6.70	7.18	3.15	3.52	4.22	4.62	5.03	5.80	6.50
	34	4.02	4.23	4.96	5.32	5.73	6.68	7.20	3.15	3.56	4.23	4.61	4.90	5.91	6.67
	35	3.73	4.18	4.95	5.30	5.66	6.65	7.34	2.60	3.52	4.26	4.57	4.99	5.87	7.44
	36	3.95	4.18	4.86	5.27	5.65	6.60	7.11	2.93	3.36	4.26	4.65	5.00	5.85	6.15
	37	3.93	4.09	4.84	5.23	5.64	6.44	6.93	2.83	3.51	4.25	4.66	5.05	5.93	7.03
	38	3.02	3.80	4.84	5.24	5.75	6.89	7.12	3.18	3.53	4.17	4.51	4.98	5.79	6.45
	39	3.75	4.08	4.91	5.29	5.77	6.63	7.00	3.11	3.50	4.15	4.50	4.88	5.87	6.27
	40	3.25	3.79	4.69	5.21	5.55	6.55	7.05	3.18	3.30	4.19	4.53	4.95	5.94	6.15
	41	3.45	3.99	4.74	5.17	5.59	6.49	6.83	3.05	3.46	4.13	4.53	4.90	5.91	6.72
	42	3.09	3.85	4.79	5.15	5.62	6.56	7.18	3.19	3.43	4.05	4.49	4.91	6.16	7.00
	43	3.67	3.87	4.72	5.13	5.50	6.68	7.28	3.00	3.36	4.15	4.53	4.85	5.67	6.45
	44	3.29	3.85	4.74	5.17	5.61	6.38	6.77	2.78	3.30	4.09	4.52	4.92	6.05	6.26
	45	3.17	3.78	4.80	5.22	5.61	6.36	6.72	2.95	3.29	4.12	4.48	4.86	5.79	6.26
	46	2.98	3.44	4.59	5.04	5.52	6.49	7.15	2.70	2.98	4.04	4.48	4.87	5.89	6.38
	47	2.85	3.55	4.69	5.16	5.53	6.41	6.95	2.90	3.25	4.04	4.46	4.88	5.90	6.53
	48	3.27	3.68	4.71	5.13	5.58	6.61	7.04	2.90	3.27	4.08	4.46	4.84	5.69	6.37
	49	2.83	3.53	4.59	5.03	5.49	6.28	6.60	2.55	2.94	4.06	4.50	4.92	5.91	6.40
	50	3.20	3.52	4.62	5.16	5.48	6.40	6.66	2.80	3.25	4.09	4.50	4.90	5.99	7.07
	51	3.02	3.37	4.58	5.02	5.50	6.55	6.88	2.73	3.12	4.07	4.51	4.97	5.83	6.34
	52	3.24	3.62	4.56	5.04	5.47	6.39	7.01	2.88	3.40	4.07	4.55	4.92	5.88	6.56
	53	3.22	3.82	4.54	4.97	5.42	6.38	6.90	2.91	3.37	4.07	4.54	4.98	5.94	6.94
	54	3.02	3.53	4.52	4.92	5.37	6.36	6.79	2.97	3.26	4.11	4.57	4.96	5.91	6.54
	55	3.24	3.66	4.53	4.90	5.35	6.14	6.48	2.58	3.06	4.11	4.54	4.99	6.09	6.81
	56	2.67	3.26	4.47	4.84	5.32	6.20	6.56	3.18	3.45	4.14	4.55	4.99	5.90	6.44
	57	2.50	2.69	4.17	4.79	5.26	6.27	6.60	2.64	2.97	3.99	4.37	4.85	5.96	6.78

	58	2.48	2.66	4.14	4.71	5.26	5.93	6.04	2.59	3.30	4.03	4.42	4.87	5.75	6.16
	59	2.51	3.11	4.47	4.90	5.35	6.03	6.72	2.71	3.08	3.96	4.43	4.80	5.72	5.97
	60	2.69	3.22	4.47	4.90	5.31	5.94	6.32	2.79	3.11	4.04	4.44	4.88	5.83	6.63
	61	2.26	2.78	4.14	4.76	5.17	6.04	6.26	2.82	3.19	3.98	4.42	4.88	5.86	6.50
	62	2.32	2.41	4.13	4.71	5.21	6.62	7.30	2.92	3.21	3.98	4.39	4.86	5.72	6.41
	63	2.25	2.36	4.00	4.51	5.15	6.14	6.50	2.55	3.12	3.97	4.42	4.92	5.96	7.06
	64	2.37	2.93	4.16	4.64	5.11	6.02	6.40	2.07	2.90	4.03	4.44	4.83	5.78	6.18
	65	2.56	2.94	4.01	4.61	5.10	5.84	6.60	2.85	3.19	3.98	4.40	4.79	5.78	6.50
	66	2.41	2.93	4.10	4.60	5.07	6.03	6.33	2.74	3.02	4.01	4.40	4.82	5.82	6.42
	67	2.19	2.60	3.75	4.52	5.01	6.20	6.92	2.59	3.31	3.97	4.39	4.81	5.60	5.96
	68	2.08	2.51	4.02	4.50	5.01	5.85	6.23	2.65	3.06	3.93	4.37	4.77	5.75	6.14
	69	2.50	2.63	3.65	4.34	4.89	5.82	6.15	2.02	2.62	3.83	4.29	4.75	5.52	6.05
	70	2.35	2.61	3.74	4.24	4.67	5.56	5.99	2.75	3.22	3.93	4.27	4.76	5.63	6.16
FT4 (pM)	22	10.86	14.48	16.49	18.44	20.21	24.12	25.29	10.45	11.61	15.21	16.93	18.62	21.91	22.72
	23	13.20	14.09	16.94	18.62	20.30	23.39	25.53	11.07	12.30	16.01	17.59	19.26	21.65	24.76
	24	12.30	13.47	16.96	18.60	20.26	24.14	24.74	10.34	12.44	15.70	17.19	18.73	22.80	24.11
	25	12.72	14.07	16.80	18.50	20.11	23.98	25.18	11.16	12.31	15.53	17.11	18.94	22.77	25.20
	26	12.55	14.64	16.92	18.42	20.26	23.97	26.02	10.76	12.27	15.53	17.01	18.69	22.68	24.80
	27	12.42	13.99	16.68	18.40	20.04	23.52	25.30	9.52	12.04	15.24	16.66	18.46	22.96	25.10
	28	12.87	13.65	16.82	18.41	20.07	24.03	25.63	10.13	11.63	15.44	16.87	18.78	22.34	25.70
	29	12.96	14.16	16.64	18.03	19.75	23.90	26.22	12.21	13.09	15.45	16.69	18.32	22.64	24.40
	30	12.53	14.08	16.78	18.23	20.01	23.92	25.98	11.22	12.34	14.97	16.51	18.22	23.13	27.05
	31	13.43	14.12	16.69	18.01	19.65	23.77	25.73	10.25	12.19	15.23	16.76	18.53	23.31	25.47
	32	12.30	13.37	16.49	18.04	19.75	24.05	26.18	10.79	12.19	14.87	16.69	18.27	21.60	25.50
	33	12.62	13.42	16.31	18.05	19.70	23.16	25.29	11.74	12.64	15.05	16.44	18.01	21.99	25.35
	34	12.74	13.52	16.17	17.81	19.48	22.97	25.03	11.72	12.67	15.08	16.53	17.97	22.54	25.23
	35	12.03	12.88	16.18	17.76	19.20	22.80	24.87	10.50	12.54	15.24	16.79	18.39	22.13	24.69
	36	12.25	13.13	15.97	17.62	19.34	23.38	24.42	11.49	12.22	15.15	16.60	18.14	21.31	24.37
	37	12.44	13.73	16.27	17.66	19.34	23.05	25.89	10.03	11.68	14.87	16.28	17.83	21.73	23.54
	38	11.90	13.24	16.00	17.62	19.33	23.56	24.69	11.33	12.18	14.70	16.32	17.89	21.72	24.33
	39	11.74	12.45	15.67	17.21	18.95	22.80	24.71	9.99	11.46	14.41	15.80	17.22	21.20	22.84
	40	8.77	12.40	15.90	17.65	19.23	23.18	24.85	11.49	12.20	14.64	15.90	17.41	23.19	25.04
	41	11.86	13.41	15.97	17.45	18.76	21.89	23.77	8.27	11.93	14.47	15.98	17.76	22.89	24.63
	42	9.77	12.72	15.65	17.27	18.87	22.90	25.34	8.52	11.20	14.69	15.91	17.85	21.28	24.63
	43	8.33	12.02	15.54	17.20	18.87	22.88	26.86	10.09	11.80	14.69	16.21	17.53	21.15	21.83
	44	10.75	12.13	15.86	17.36	18.98	22.93	24.70	9.88	12.00	14.59	16.25	17.85	21.05	22.15
	45	11.08	12.36	15.51	17.39	18.90	22.41	24.74	9.02	11.40	14.49	16.16	17.99	22.48	24.49
	46	11.06	12.58	15.58	17.07	18.94	22.58	24.47	10.48	11.47	14.47	15.93	17.46	22.20	23.41
	47	11.07	12.08	15.58	17.24	19.25	22.11	23.89	10.41	11.54	14.17	15.64	17.41	21.75	23.45
	48	11.55	12.61	15.62	17.24	18.83	22.15	24.13	9.77	11.61	14.30	16.01	17.52	21.42	23.91
	49	10.30	12.53	15.34	16.85	18.48	23.01	24.82	8.77	11.02	14.22	15.89	17.88	21.76	23.95
	50	10.11	12.48	15.43	17.12	18.59	22.22	23.64	9.77	10.77	14.63	15.81	17.46	22.50	24.18
	51	11.56	12.60	15.65	16.94	18.50	22.48	24.32	9.40	11.85	14.51	15.90	17.79	21.55	23.54
	52	10.67	11.90	14.95	16.58	18.40	22.88	24.87	10.59	11.64	14.56	16.19	17.94	22.13	24.56
	53	10.09	11.94	15.07	16.83	18.57	22.01	24.07	9.55	11.31	14.79	16.36	18.30	22.68	26.04
	54	10.26	11.90	15.18	17.13	18.56	22.55	24.48	9.48	11.90	14.78	16.19	17.76	22.27	24.03
	55	10.76	11.70	15.15	16.70	18.29	21.95	24.05	10.33	11.99	14.71	16.35	18.04	22.16	25.05

	56	9.49	11.59	14.96	16.88	18.32	22.56	26.75	10.71	11.96	14.93	16.48	18.29	23.35	26.49
	57	10.94	12.49	14.72	16.50	18.00	20.82	21.45	9.66	12.09	14.61	16.00	17.74	22.19	24.29
	58	9.45	11.37	14.95	16.69	18.56	22.49	24.06	10.38	11.76	14.84	16.57	18.29	22.20	26.59
	59	8.71	11.71	14.75	16.33	18.35	23.23	25.53	10.98	11.59	14.75	16.36	17.81	22.34	24.52
	60	9.83	11.41	14.90	16.58	18.28	22.80	23.75	10.91	11.96	14.87	16.25	18.33	22.41	23.97
	61	10.95	12.72	15.18	17.06	18.77	23.35	25.19	10.92	12.30	14.83	16.26	18.09	21.51	25.80
	62	9.14	11.00	14.75	16.54	18.43	21.19	21.69	11.28	11.88	14.76	16.25	17.84	22.12	24.39
	63	8.49	11.29	14.52	16.49	18.30	23.37	24.97	10.19	12.12	14.93	16.47	18.27	22.01	23.84
	64	11.07	11.65	14.87	16.41	18.13	22.67	24.54	7.63	10.82	14.65	16.23	18.02	22.49	24.12
	65	9.35	11.53	14.95	16.51	18.27	21.96	22.89	11.12	12.30	14.78	16.35	18.20	22.76	25.90
	66	5.45	10.82	14.77	16.77	19.03	22.71	25.47	10.10	11.98	14.82	16.50	18.44	21.93	25.55
	67	8.62	10.02	14.89	16.83	19.17	24.22	26.06	10.61	12.04	14.83	16.42	18.21	22.09	24.08
	68	9.56	10.62	14.36	16.08	18.01	22.94	24.31	10.86	12.33	14.74	16.43	18.27	22.67	23.80
	69	10.93	11.96	14.78	16.49	18.03	22.38	24.71	11.43	12.37	14.80	16.48	18.00	22.30	23.88
	70	9.62	11.28	14.90	16.75	18.92	22.32	23.83	10.81	11.73	14.93	16.27	17.96	21.99	23.34
TSH (mIU/L)	22	0.60	0.86	1.60	2.17	3.01	5.07	5.89	0.47	0.83	1.56	2.15	2.90	4.95	5.49
	23	0.52	0.80	1.56	2.12	2.92	4.93	5.41	0.01	0.48	1.55	2.27	3.13	5.20	5.76
	24	0.49	0.80	1.62	2.15	2.96	5.03	5.67	0.06	0.75	1.62	2.28	3.12	5.24	5.90
	25	0.58	0.80	1.55	2.12	2.94	5.04	5.62	0.34	0.72	1.64	2.30	3.15	5.30	6.02
	26	0.60	0.80	1.56	2.12	2.88	4.89	5.50	0.32	0.71	1.60	2.26	3.07	5.22	6.02
	27	0.45	0.80	1.60	2.19	2.97	4.99	5.78	0.08	0.67	1.66	2.31	3.30	5.70	6.32
	28	0.52	0.78	1.53	2.12	2.90	4.96	5.59	0.09	0.63	1.61	2.24	3.04	5.13	5.91
	29	0.54	0.80	1.54	2.11	2.91	4.96	5.55	0.29	0.66	1.62	2.33	3.13	5.27	6.09
	30	0.45	0.77	1.55	2.10	2.88	4.87	5.64	0.07	0.63	1.62	2.28	3.27	5.32	6.32
	31	0.50	0.80	1.54	2.10	2.83	4.67	5.38	0.08	0.49	1.61	2.27	3.16	5.48	5.90
	32	0.39	0.85	1.47	2.05	2.75	4.62	5.55	0.38	0.76	1.73	2.26	3.15	5.27	5.89
	33	0.49	0.85	1.47	2.06	2.77	4.86	5.36	0.15	0.60	1.71	2.30	3.25	5.45	6.28
	34	0.43	0.70	1.50	2.09	2.92	5.03	6.00	0.28	0.68	1.66	2.38	3.37	5.68	6.44
	35	0.43	0.78	1.51	2.14	2.93	4.94	6.23	0.20	0.63	1.58	2.31	3.14	5.42	6.03
	36	0.41	0.74	1.44	2.00	2.66	4.48	4.84	0.23	0.81	1.66	2.31	3.16	5.28	5.66
	37	0.23	0.71	1.45	2.09	2.90	5.06	5.88	0.19	0.64	1.64	2.19	3.00	5.13	5.46
	38	0.07	0.55	1.41	1.97	2.80	4.73	5.73	0.09	0.44	1.48	2.21	3.27	5.51	6.05
	39	0.38	0.75	1.46	2.01	2.82	4.82	6.39	0.01	0.55	1.70	2.36	3.24	5.48	6.04
	40	0.37	0.71	1.47	2.00	2.81	4.81	5.84	0.10	0.59	1.57	2.25	3.36	5.90	6.22
	41	0.35	0.71	1.50	2.10	2.85	4.86	5.62	0.20	0.39	1.49	2.23	3.19	5.47	5.88
	42	0.39	0.62	1.39	1.94	2.74	4.63	5.75	0.17	0.53	1.72	2.37	3.28	5.39	6.12
	43	0.29	0.77	1.41	1.99	2.86	4.96	6.03	0.22	0.57	1.66	2.43	3.45	5.49	6.41
	44	0.38	0.69	1.40	1.98	2.78	4.71	5.15	0.14	0.81	1.69	2.42	3.45	5.60	6.13
	45	0.36	0.74	1.43	2.07	2.97	5.15	5.75	0.10	0.44	1.57	2.54	3.57	5.75	6.26
	46	0.40	0.72	1.43	2.00	2.80	4.85	5.73	0.23	0.81	1.69	2.43	3.65	6.03	6.65
	47	0.39	0.71	1.48	1.92	2.66	4.46	4.80	0.23	0.65	1.70	2.57	3.41	5.82	6.50
	48	0.20	0.69	1.38	1.89	2.60	4.45	4.77	0.49	0.84	1.93	2.72	3.74	6.13	6.91
	49	0.31	0.62	1.48	2.01	2.74	4.64	5.56	0.10	0.46	1.76	2.65	3.70	6.15	6.62
	50	0.19	0.68	1.45	2.04	2.93	5.07	5.63	0.06	0.40	1.84	2.54	3.68	6.27	6.82
	51	0.32	0.69	1.55	2.14	2.82	4.55	5.17	0.06	0.39	1.77	2.67	3.84	6.29	6.93
	52	0.21	0.66	1.56	2.02	2.75	4.45	5.40	0.07	0.40	1.65	2.59	3.77	5.90	6.72
	53	0.52	0.79	1.52	2.04	2.90	4.98	5.49	0.07	0.41	1.77	2.68	3.90	6.20	6.67

54	0.32	0.70	1.44	2.17	3.05	5.30	5.87	0.26	0.74	1.91	2.80	3.84	6.29	6.97
55	0.36	0.66	1.51	2.15	3.07	5.37	6.30	0.14	0.42	1.83	2.76	3.91	6.40	7.26
56	0.26	0.60	1.50	2.13	3.02	4.63	5.92	0.03	0.42	1.83	2.77	3.95	6.62	7.09
57	0.07	0.61	1.38	1.97	2.87	4.44	5.13	0.26	0.78	1.84	2.77	4.06	6.40	6.93
58	0.29	0.65	1.59	2.29	3.39	5.54	6.03	0.18	0.44	1.89	2.75	4.03	6.28	6.85
59	0.29	0.67	1.48	2.04	3.06	4.83	5.43	0.18	0.50	1.98	2.82	3.87	6.19	6.73
60	0.15	0.67	1.39	2.04	2.90	4.79	4.99	0.19	0.75	1.84	2.92	3.96	6.41	6.78
61	0.15	0.52	1.38	2.03	2.67	4.54	4.80	0.07	0.54	1.89	2.88	4.18	6.49	6.83
62	0.31	0.49	1.57	2.17	3.08	5.27	5.75	0.12	0.56	1.91	2.88	4.18	6.21	7.19
63	0.22	0.44	1.52	2.14	3.06	5.35	5.87	0.02	0.38	1.83	2.73	3.94	6.45	7.10
64	0.21	0.46	1.44	2.04	3.14	5.18	6.26	0.33	0.65	1.82	2.80	4.01	6.58	7.14
65	0.34	0.55	1.46	2.05	2.87	4.79	5.59	0.28	0.78	1.92	2.74	3.75	6.16	7.02
66	0.26	0.59	1.44	1.95	2.82	4.82	5.13	0.23	0.61	1.92	2.86	4.05	6.33	7.09
67	0.15	0.45	1.43	2.01	3.21	4.97	6.14	0.18	0.65	2.00	2.91	4.04	6.45	6.87
68	0.38	0.59	1.49	2.08	3.33	5.26	5.62	0.20	0.67	2.12	3.09	4.34	6.42	6.87
69	0.33	0.50	1.42	1.92	2.54	3.96	4.10	0.32	0.61	1.86	2.78	3.89	6.36	7.14
70	0.37	0.53	1.44	2.06	2.96	5.06	5.67	0.28	0.69	1.86	2.70	3.79	6.33	6.98

Abbreviation: T3, triiodothyronine; T4, thyroxine; FT3, free triiodothyronine; FT4, free thyroxine; TSH, thyroid-stimulating hormone.

Figure S6. The distribution histograms of serum T3 over age and sex

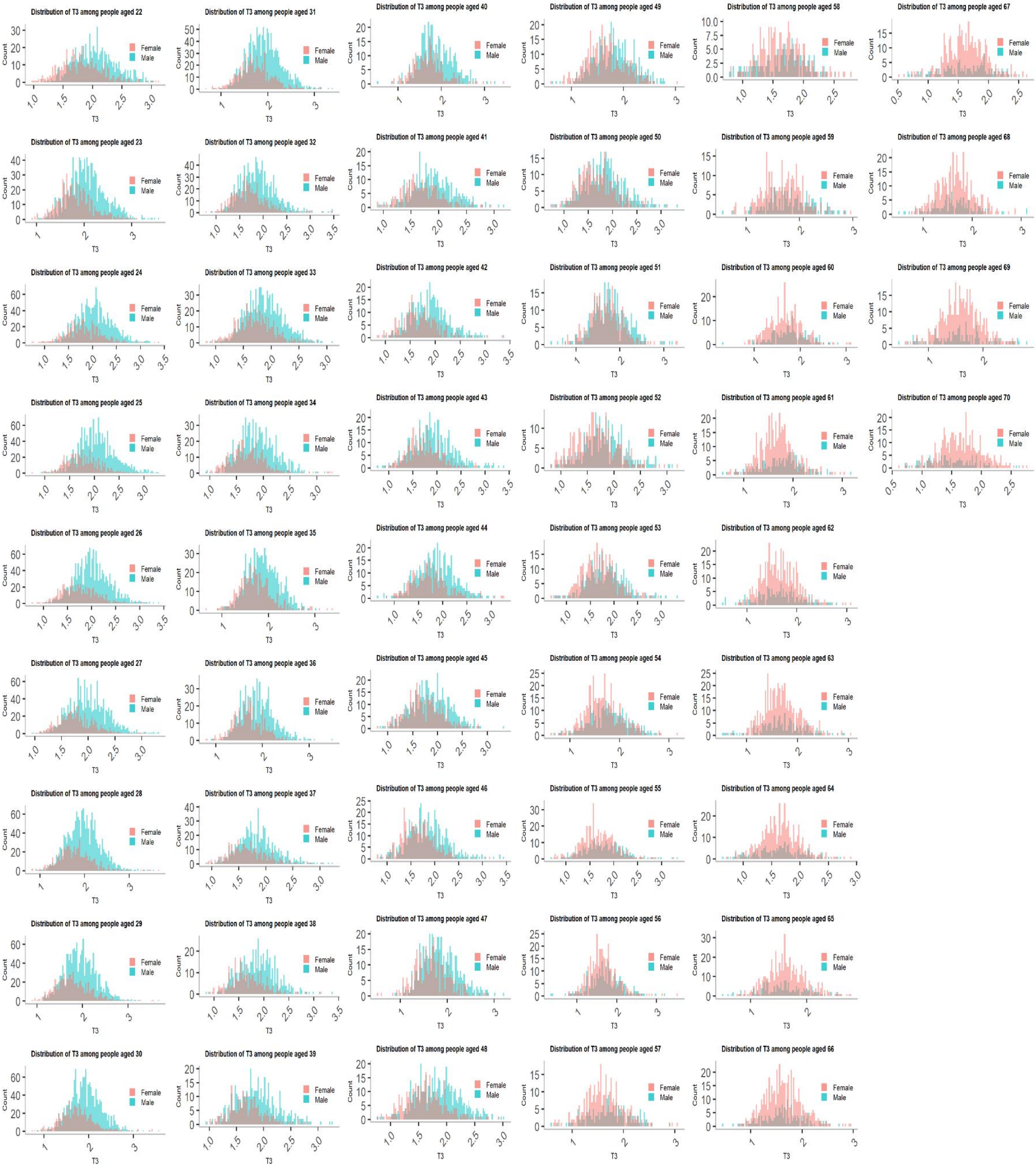

Figure S7. The distribution histograms of serum T4 over age and sex

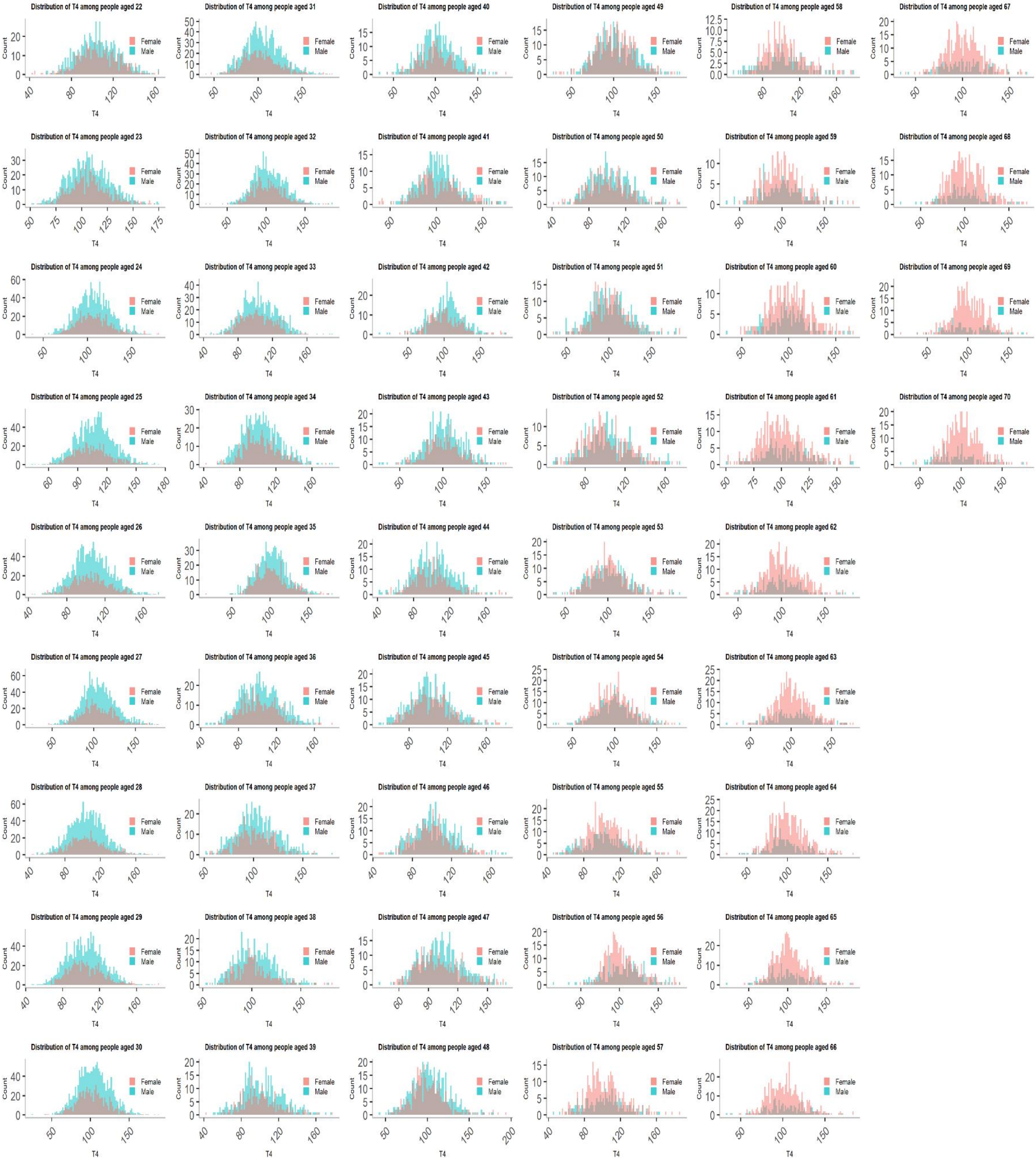

Figure S8. The distribution histograms of serum FT3 over age and sex

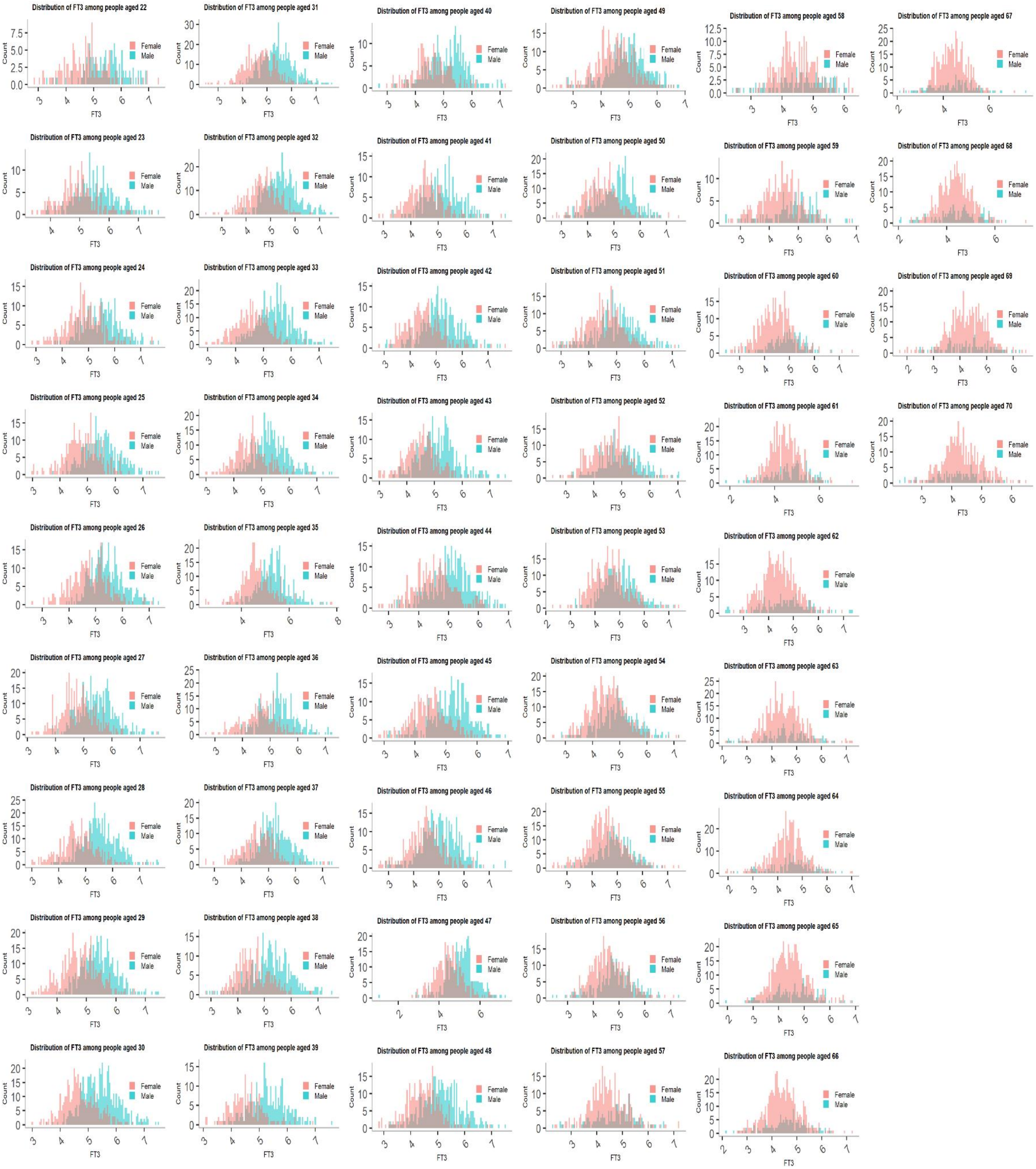

Figure S9. The distribution histograms of serum FT4 over age and sex

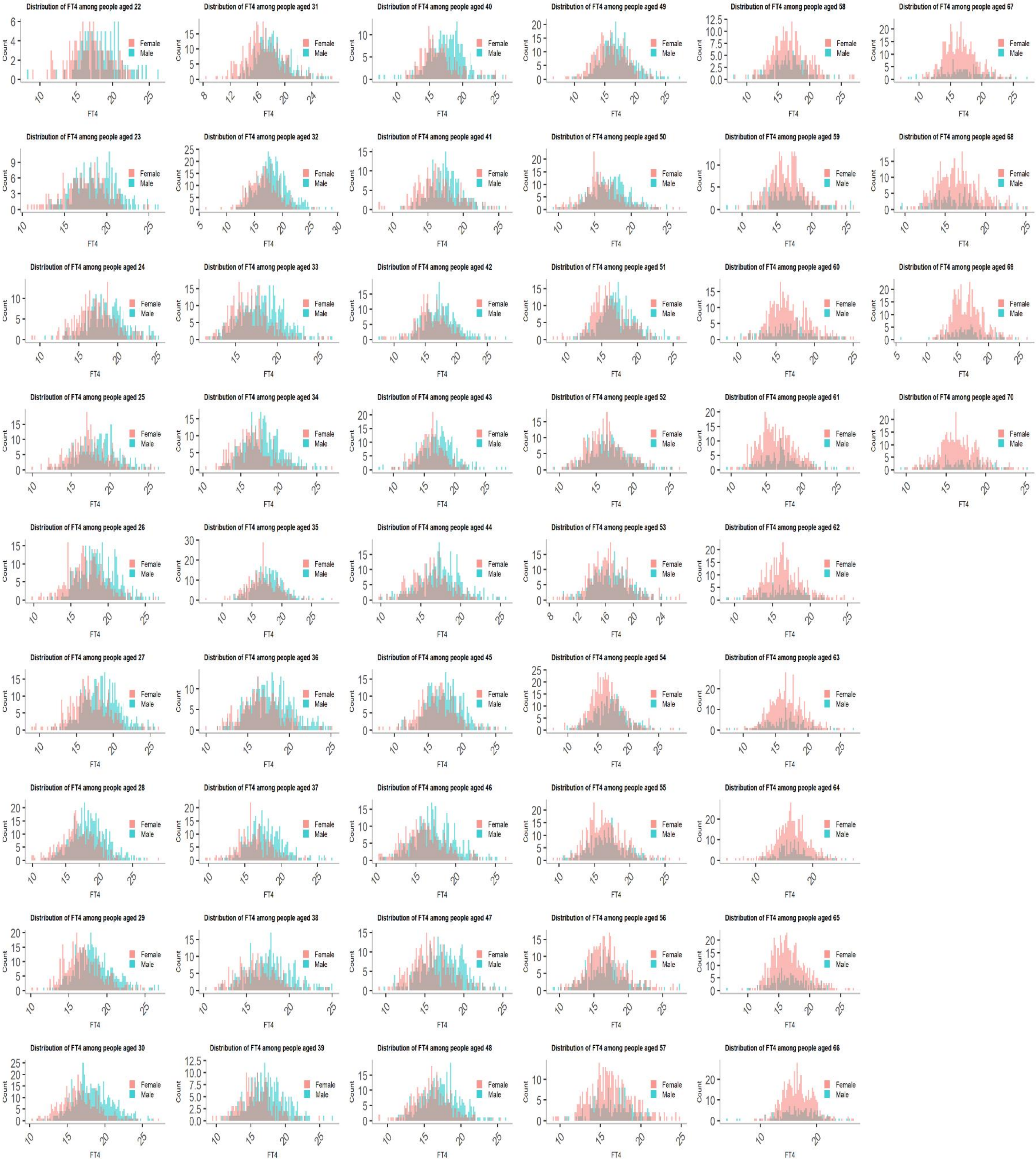

Figure S10. The distribution histograms of serum TSH over age and sex

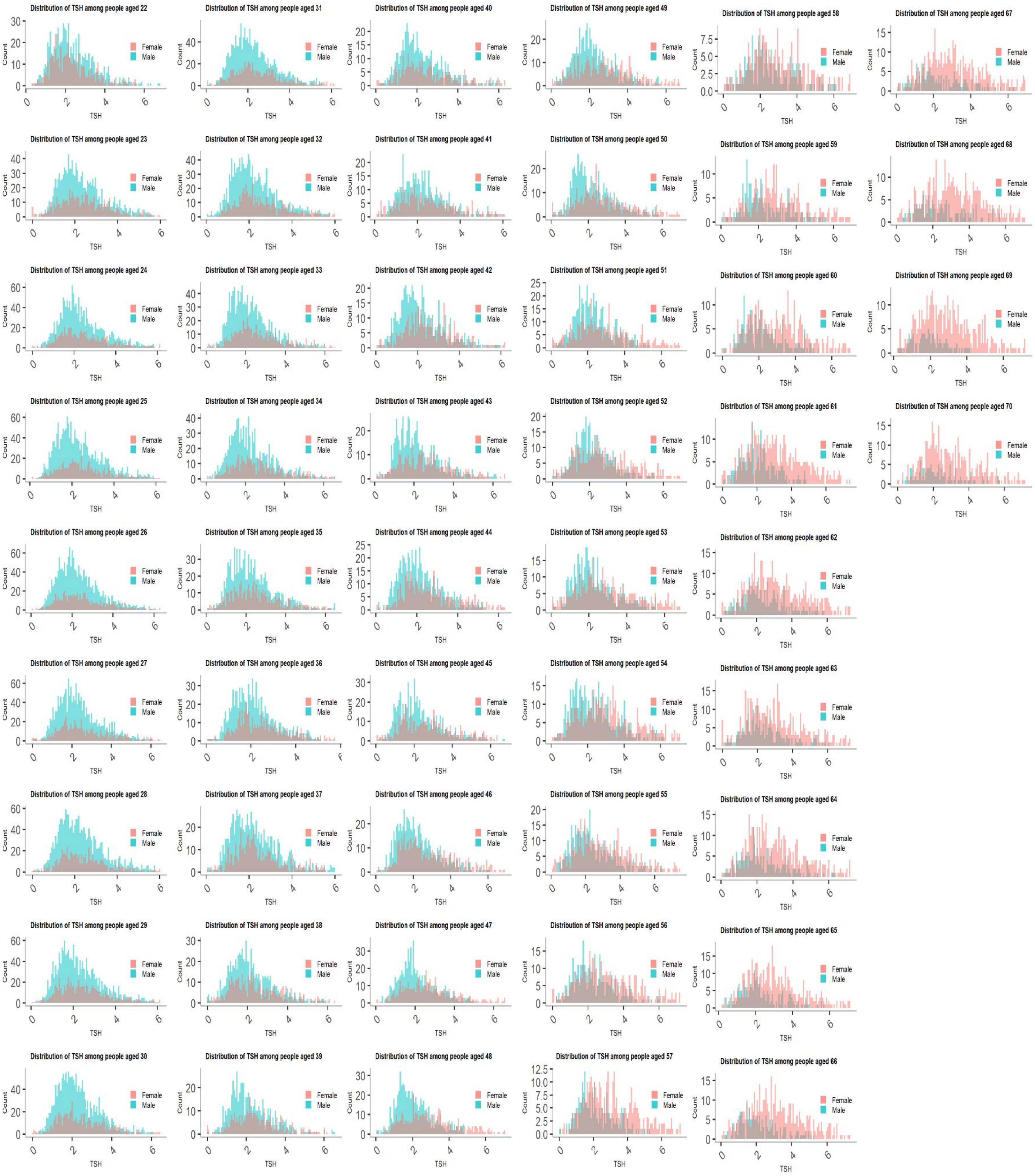

Supplement: Supplementary file 1 [file DataSheet_1.pdf]
